# Evaluation of the Luminex xTAG Respiratory Viral Panel FAST v2 assay for detection of multiple respiratory viral pathogens in nasal and throat swabs in Vietnam

**DOI:** 10.12688/wellcomeopenres.12429.2

**Published:** 2018-04-30

**Authors:** Vu Thi Ty Hang, Nguyen Thi Han Ny, Tran My Phuc, Pham Thi Thanh Tam, Dang Thao Huong, Ho Dang Trung Nghia, Nguyen Tran Anh Vu, Pham Thi Hong Phuong, Nguyen Van Xang, Nguyen Dong, Pham Nhu Hiep, Nguyen Van Hung, Tran Tinh Hien, Maia Rabaa, Guy E. Thwaites, Stephen Baker, Le Van Tan, H.Rogier van Doorn, VIZIONS consortium

**Affiliations:** 1 Hospital for Tropical Diseases, Wellcome Trust Major Overseas Programme, Oxford University Clinical Research Unit, Ho Chi Minh City, Vietnam; 2 Dong Thap General Hospital, Dong Thap, Vietnam; 3 Khanh Hoa General Hospital, Khanh Hoa, Vietnam; 4 Hue Central Hospital, Thua Thien - Hue, Vietnam; 5 Dak Lak General Hospital, Dak Lak, Vietnam; 6 Centre for Tropical Medicine, Nuffield Department of Clinical Medicine, Oxford University, Oxford, UK; 7 The London School of Hygiene and Tropical Medicine, London, UK; 8 National Hospital for Tropical Diseases, Wellcome Trust Major Overseas Programme, Oxford University Clinical Research Unit, Hanoi, Vietnam

**Keywords:** Luminex, RVP FAST v2, Vietnam

## Abstract

**Background**: Acute respiratory infections (ARI) are among the leading causes of hospitalization in children ≤5 years old. Rapid diagnostics of viral pathogens is essential to avoid unnecessary antibiotic treatment, thereby slowing down antibiotic-resistance. We evaluated the diagnostic performance of the Luminex xTAG Respiratory Viral Panel FAST v2 against viral specific PCR as reference assays for ARI in Vietnam.

**Methods**: Four hundred and forty two nose and throat swabs were collected in viral transport medium, and were tested with Luminex xTAG Respiratory Viral Panel FAST v2. Multiplex RT-PCR and single RT-PCR were used as references.

**Results**: Overall, sensitivity of the Luminex against reference assays was 91.8%, 95% CI 88.1-94.7 (270/294), whilst 112/6336 (1.8%, 95% CI, 1.4-2.1) of pathogens were detected by the Luminex, but not by reference assays. Frequency of pathogens detected by Luminex and reference assays was 379 and 292, respectively. The diagnostic yield was 66.7% (295/442, 95%CI 62.1-71.1%) for the Luminex assay and 54.1% (239/442, 95% CI, 49.3-58.8%) for reference assays. The Luminex kit had higher yields for all viruses except influenza B virus, respiratory syncytial virus, and human bocavirus. High agreements between both methods [mean (range): 0.91 (0.83-1.00)] were found for 10/15 viral agents.

**Conclusions**: The Luminex assay is a high throughput multiplex platform for rapid detection of common viral pathogens causing ARI. Although the current high cost may prevent Luminex assays from being widely used, especially in limited resource settings where ARI are felt most, its introduction in clinical diagnostics may help reduce unnecessary use of antibiotic prescription.

## Introduction

Acute respiratory infections (ARI) are the leading cause of morbidity and mortality in infants and children worldwide, especially in Southeast Asia (including Vietnam) and Africa ^1, 2^. In Vietnam, there is a high burden of ARI in children in the first year of life, who are more likely to be admitted to intensive care and have a longer hospital stay than children with other infectious diagnoses ^3^.

Viruses are the most common causes of ARI ^4– 8^. Rapid identification of causative agents is therefore of clinical and public health significance, and may reduce the widespread inappropriate use of antibiotics.

The advances in molecular diagnostics have provided powerful means for detection of viruses in terms of sensitivity, specificity and turnaround time ^5, 9– 12^. The introduction of multiplex PCR for detection of a panel of respiratory pathogens enables faster results, higher throughput and lower cost ^13, 14^.

The Luminex xTAG Respiratory Viral Panel (RVP) FAST v2 assay (Luminex Molecular Diagnostics, Toronto, ON, Canada) is a qualitative multiplex molecular diagnostic assay for simultaneous detection of 19 viral types and subtypes within two hours in a single reaction. It was approved in September 2012 by the US Food and Drug Administration (FDA). Previous reports have shown that the sensitivity of Luminex RVP FAST system varied between settings, while the specificity was consistently high ^15– 19^. Here, we aimed to evaluate the diagnostic performance of the Luminex xTAG RVP FAST v2 (hereafter called Luminex) against a combination of a number of published reference assays on clinical specimens collected from patients with ARI from four provincial hospitals in Vietnam.

## Methods

### Study samples and study procedure

This is a retrospective study. Respiratory samples were derived from the Vietnam initiative on Zoonotic infections (VIZIONS) study ^20^. This study was approved by the local ethical committee and the Oxford Tropical Research Ethics Committee (OxTREC Approval No. 15–12). The Institutional Review Boards of the hospital sites (Daklak, Dong Thap, Hue, and Khanh Hoa) submitted the official document approvals to the Hospital for Tropical Diseases (HTD) local ethics committee. The HTD then gave ethical approval (approval no. CS/ND/13/28). Written informed consent was obtained from all patients or from parents/legal guardians if the patient was a child prior to enrolment into the study. In and outpatients with a clinical diagnosis of ARI were recruited from four provincial hospitals (in Cao Lanh, Dong Thap; Buon Me Thuot, Dak Lak; Nha Trang, Khanh Hoa; and Hue). The study inclusion criteria consisted of fever or history of fever of less than 7 days and respiratory symptoms as the chief complaint. On the day of enrollment, nose and throat swabs were collected from each patient in separate tubes containing 1ml of sterile viral transport medium (VTM, including MEM Hanks: 500ml, gelatin 5%: 50ml and antibiotics (antbiotics: penicillin 100UI/mlVTM + streptomycin 100mg/ml VTM : 5ml and Amphotericin B 250ug/ml: 3ml). Samples were stored at -80°C and transported in batches on dry ice to the laboratory of Oxford University Clinical Research Unit (OUCRU), Ho Chi Minh City, where the nose and throat swabs were pooled for subsequent analysis as per study protocols. Four hundred forty-two samples were simultaneously tested with the Luminex assay and reference assays.

### Nucleic acid extraction

Equal volumes of VTM from nose and throat swabs were pooled and subjected to total nucleic acid extraction after addition of internal control (EAV - equine arteritis virus for reference assays and bacteriophage MS2 for the Luminex assay) using the MagNApure 96 platform (Roche Diagnostics, Mannheim, Germany), according to the manufacturer’s instructions. Extracted nucleic acids were eluted in 50 ul of elution buffer and stored at -80°C for further analyses.

### Luminex xTAG RVP FAST v2 set

The kits were provided for free by the Luminex company. The Luminex assay includes reagents to detect Influenza A virus (InfA: generic, H1N1 (1977), H1N1pdm09, H3N2), Influenza B virus (InFB), Respiratory Syncytial Virus A & B (RSVA, RSVB), enteroviruses including rhinoviruses (ENT/Rhi), human parainfluenza viruses 1–4 (PIV1-4), human metapneumovirus (hMPV), adenovirus (ADV), human coronavirus NL63 (hCoV NL63), hCoV HKU1, hCoV 229E, hCoV OC43, and human bocavirus (hBoV). Bacteriophage lambda was included in every run to control the amplification and assay performance. The assay comprised a PCR amplification and hybridization step, and was performed according to the manufacturer’s instructions. Signal acquisition presented as MFI (median fluorescence intensity) was done on the Luminex MAGPIX instrument. Data was read and reported by the TDAS RVP FAST software, version 2.2.

### Reference assays

The reference assays included an RT-PCR for RSVA and RSVB ^21^, the CDC RT-PCR obtained by protocol transfer agreement with the US CDC for universal detection of influenza A virus, influenza A virus subtype H1 (1977), influenza A virus subtype H3, and InFB [ Table 1, *CDC Realtime RTPCR (rRTPCR) Protocol for Detection and Characterization of Influenza, Revised April 4, 2006*]; and the 4-tube real time multiplex RT-PCR ^22^. Because the influenza A viruses, InfB and RVS targets were tested in the above specific RT-PCRs, they were not tested in the 4-tube assay. Detection of Influenza A virus H1N1pdm09 was performed by an in-house RT-PCR assay using primer set targeting HA (haemagglutinin) gene segment 4 (forward primer, 5’-GTTACCCAGGAGATTTCATCGA-3’; reverse primer, 5’-CATGCTGCCGTTACACCTTTG-3’; and probe, 5’-FAM-AAGTTCATGGCCCAATCATGACTCGA-BHQ1-3’ [FAM, 6-carboxyfluorescein, BHQ1, black hole quencher 1]). The reference was considered to be positive if any one of the reference assays was positive.

**Table 1.** Primers and probe sequences of CDC influenza A/B virus PCR.

Primer name	Oligo sequence (5′>3′)
FluA Forward	GACCRATCCTGTCACCTCTGAC
FluA Reverse	AGGGCATTYTGGACAAAKCGTCTA
FluA probe	FAM-TGCAGTCCTCGCTCACTGGGCACG-BHQ1
1977 seasonal H1 Forward	AACTACTACTGGACTCTGCTGGAA
1977 seasonal H1 Reverse	CCATTGGTGCATTTGAGGTGATG
1977 Seasonal H1 Probe ^1^	FAM-TGAYCCAAAGCC”T” (BHQ1)CTACTCAGTGCGAAAGC
FluA H3 Forward	AAGCATTCCYAATGACAAACC
FluA H3 Reverse	ATTGCRCCRAATATGCCTCTAGT
FluA H3 Probe	FAM-CAGGATCACATATGGGSCCTGTCCCAG-BHQ1
FluB Forward	TCCTCAACTCACTCTTCGAGCG
FluB Reverse	CGGTGCTCTTGACCAAATTGG
FluB probe	FAM-CCAATTCGAGCAGCTGAAACTGCGGTG-BHQ1

Note: FAM: 6-carboxyfluorescein (FAM); BHQ1: Blackhole Quencher 1. 1: Taqman ^®^ probe is internally quenched at a modified “T” residue with BHQ1.

### Data analysis and statistical analysis

Performance of the Luminex assay was evaluated as diagnostic yields and sensitivity against reference assays with 95% confidence interval using 2x2 tables. The calculation of these was performed with Intercooled Stata 9.2 (Stata, College station, TX, USA). Agreement between the Luminex and reference assays was determined by Kappa statistic using SPSS version 23 (IBM Corp. SPSS Statistic, NY, USA). McNemar test (SPSS version 23) was used to examine whether there was any difference of diagnostic rates of individual viruses between the Luminex and reference assays. Statistical significance was set at P<0.05.

## Results

### Baseline results

A total of 442 samples collected between November 2012 and April 2014 were analysed for the evaluation of the Luminex assay. Three hundred forty-eight samples were from children (≤15 years old) and 94 samples from adults. The male/female ratio was 0.62 (273): 0.38 (169). The median age of children was 1 year (Interquartile range, IQR: 1, 2) and of adults 46 years (IQR: 34, 72). Overall, 302 specimens (68.3%) were positive by either Luminex or reference assays or both. One hundred forty samples (31.7%) remained undiagnosed. ENT/Rhi was the most frequently detected pathogen by the two techniques (159 over 403 total count of all pathogens, 40%), followed by hBoV (n = 45, 11%) and PIV3 (n = 42, 10%). Less frequently detected were ADV (n = 32, 8%), hMPV (n = 29, 7%), InFA (n = 22, 6%), hCoV (n = 22, 6%), RSV A and B (n = 21, 5%), PIV4 (n = 12, 3%), InFB (n = 10, 2.5%), PIV1 (n = 6, 1%) and PIV2 (n = 3, 0.5%).

### Comparison of the Luminex xTAG RVP FAST v2 test and references

**Figure 1.**
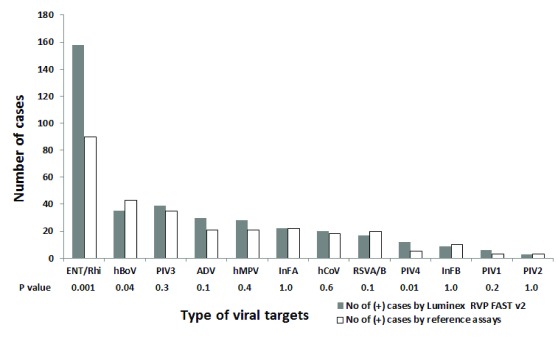
Comparison of count of individual pathogens detected by the Luminex RVP FAST v2 and reference assays.

Diagnostic yields of Luminex and reference assays were 66.7% (295/442, 95% CI, 62.1-71.1%) and 54.1% (239/442, 95% CI, 49.3-58.8%), respectively. The frequency of pathogens detected by Luminex and reference assays was 379 and 292, respectively. Shown in Figure 1 is number of cases that were positive for individual viruses detected by the Luminex and reference assays. The Luminex assay had a higher detection rate for most viruses, but significant differences were seen in ENT/Rhi (99% versus 57%, *P*=0.001, McNemar test) and PIV4 (100% versus 42%, *P*=0.01, McNemar test). In contrast, for hBoV, RSV and InFB, the reference assays had higher detection rates (78% versus 96%, *P*=0.04; 90% versus 100%, *P*>0.05; and 90% versus 100%, *P*>0.05; respectively). Regarding mixed-infection, the Luminex assay also detected more co-infections compared to the reference assays: 68 versus 47. The maximum number of pathogens detected in a single patient was 4. Parechoviruses were detected in four samples by the reference assays, but were not included in the Luminex assay. HCoV were not subtyped by reference assays.

### Evaluation of the test performance and agreement between assays

Table 2 shows the diagnostic performance of the Luminex assay on clinical swabs against reference assays. Overall, 270 pathogens [91.8% (95% CI, 88.1-94.7)] detected by reference assays were also detected by Luminex assay (true positive rate or “sensitivity” against reference assays as gold standard). There were 112 pathogens detected by Luminex but not detected by references, corresponding to an additional 1.8% (95%CI: 1.4-2.1) positives. In addition, there were 24 pathogens detected by reference assays but not detected by the Luminex, corresponding to an additional 0.3% (95% CI: 0.2-0.6) positives.

**Table 2.** Diagnostic performance of the Luminex RVP FAST v2 in comparison with reference assays.

Pathogens	+L and +ref	+ L and -ref	-L and +ref	- L and -ref	Luminex sensitivity ** (% [95% CI])	Additional Luminex positive *** (% [95% CI])	Kappa (95% CI)
ENT/Rhi	89	69	1	283	98.8 (93.9-99.9)	19.6 (15.6-24.1)	0.62 (0.54-0.69)
hBoV	33	2	10	397	76.7 (61.4-88.2)	0.5 (0.06-1.7)	0.83 (0.73-0.92)
PIV3	32	7	3	400	91.4 (76.9-98.2)	1.7 (0.6-3.5)	0.85 (0.77-0.92)
ADV	19	11	2	410	90.5 (69.6-98.8)	2.6 (1.3-4.6)	0.67 (0.53-0.80)
hMPV	20	8	1	413	95.2 (76.2-99.9)	1.9 (0.8-3.7)	0.80 (0.68-0.91)
InFA matrix *	3	0	0	439	100 (29.2-100)	0.0 (0.0-0.0)	1.00 (1.00-1.00)
H1N1pdm09	9	1	0	432	100 (66.4-100)	0.2 (0.0-1.2)	0.95 (0.85-1.05)
H3N2	10	0	0	432	100 (69.1-100)	0.0 (0.0-0.0)	1.00 (1.00-1.00)
hCoV (OC43, NL63, HKU1)	16	4	2	420	88.8 (65.3-98.6)	0.9 (0.2-2.3)	0.83 (0.71-0.94)
RSVA	9	0	1	432	100 (55.4-99.7)	0.0 (0.0-0.0)	0.95 (0.84-1.05)
RSVB	10	0	3	429	76.9 (46.2-94.9)	0.0 (0.0-0.0)	0.87 (0.73-1.00)
PIV4	5	7	0	430	100 (47.8-100)	1.6 (0.6-3.2)	0.58 (0.30-0.85)
InFB	9	0	1	432	90.0 (55.4-99.7)	0.0 (0.0-0.0)	0.94 (0.83-1.04)
PIV1	3	3	0	436	100 (29.2-100)	0.6 (0.1-1.9)	0.66 (0.30-1.01)
PIV2	3	0	0	439	100 (29.2-100)	0.0 (0.0-0.0)	1.00 (1.00-1.00)
Overall	270	112	24	6224	91.8 (88.1-94.7)	1.8 (1.4-2.1)	0.67 (0.61-0.73)

L: Luminex, ref: reference assays, * positive for M gene but negative for H1-1977, H1N1pdm09 and H3N2. ** +L and +ref/(+L and +ref )+(-L and +ref), *** +L and – ref/(+L and – ref) +(- L and - ref).

For individual targets, detection rate by Luminex was higher for 12 targets, ranging from 88.8% to 100% (ENT/Rhi, PIV3, ADV, hMPV, InFA matrix, H1N1pdm09, H3N2, hCoV, RSVA, PIV4, PIV1, PIV2), whilst it was less often positive for hBoV and RSVB and InFB (35 vs 43, 10 vs 13 and 9 vs 10, respectively). Remarkably, 43.4% (69/159) of ENT/Rhi were detected by Luminex but were negative by reference assays.

Among the 442 clinical swabs, concordance between the two techniques was noted in 372 samples and discordance was recorded in 70 samples, showing substantial agreement (overall kappa 0.67, 95%CI 0.61–0.73). Table 2 also shows the test agreement of all viral pathogens. Almost perfect agreement was recorded in 10 pathogens, hBoV, PIV3, InFA matrix, H1N1pdm09, H3N2, hCoV, RSVA, RSVB, InFB and PIV2 (kappa 0.83 – 1.00). Substantial agreement (kappa 0.62–0.80) was seen in hMPV, ENT/Rhi, ADV and PIV1, while agreement in PIV4 was moderate (kappa 0.58).

Dataset 1: Evaluation of the Luminex xTAG Respiratory Viral Panel FAST v2 assay for detection of multiple respiratory viral pathogens in nasal and throat swabs in Vietnam **Respiratory Viral Diagnostic Result from 442 patient swabs of the Luminex and reference assays.**

****
- Site: Abbreviation of provinces where hospitals involved in the study, where patients were recruited. (DlK: Daklak, DT: Dong Thap, H: Hue, and KH: Khanh Hoa)
- Gender: patient sex, 1: Male, 2: Female
- DateCollection: Date when patient samples were collected
- Flu A type (CDC): Results of influenza A and types from the reference assay: CDC Realtime RTPCR assay
- Flu B (CDC): Results of influenza B from the reference assay: CDC Realtime RTPCR assay
- RSV type: Results of RSV A and RSV B from in-house reference PCR assay
- detected by 4tube multiplexPCR: Results of other 10 viruses from reference assay: in-house 4-tube multiplex PCR
- Luminex result: Results from Luminex assay
- Diagnose: Final diagnosis based on Luminex and reference assays, it was defined “pos” if sample was positive with any of reference assays or Luminex assay, "undiagnosed” if sample was negative with all reference assays and the Luminex assay Click here for additional data file. Copyright: © 2018 Thi Ty Hang V et al. 2018

## Discussion

Fast and reliable diagnostic tests are a practical need in helping physicians to make appropriate treatment decisions and are a useful tool in research and surveillance. We report here the evaluation of a Luminex assay on respiratory swabs collected from patients admitted to four provincial hospitals in Vietnam. Lacking a true “gold standard”, we combined a number of published PCR assays as reference tests for evaluation.

Overall positivity of Luminex among reference assays was high (91.8%, CI 95% 88.1–94.7). Besides that 5 targets (ENT/Rhi , ADV, hMPV, PIV4 and PIV1) which were all more often detected by Luminex than by reference assays and were confirmed by single-RT PCR (number of +L/-ref cases ranged from 3 – 69; Table 2). This suggests that detection using Luminex is superior to reference assays for these targets.

Compared to reference assays, our study found an increased detection rate by the Luminex for most targets, and significant difference was seen in Ent/Rhi and PIV4 (+L/-ref= 69, p<0.001; and +L/-ref= 7, p=0.01, respectively; Figure 1). Especially a high number of +L/-ref for Ent/Rhi agent shows a considerable difference with other studies in previous xTAG Luminex studies ^15, 18, 23– 25^; it probably again reveals that Luminex is a strong assay in detection of this viral agent. Meanwhile, detection rate for hBoV was significantly higher in reference assays than in the Luminex (10 versus 2, p=0.04; Figure 1), which is similar to other previous studies ^15, 18^ (sensitivity of Luminex for this viral agent was rather low, 41.4% and 20.0% in these studies, respectively).

Though the Luminex assay may be of benefit to diagnostics and cost of treatment ^12, 13, 15, 17, 26^, it requires a specific instrument for detection and data acquisition. Therefore, it may not be appropriate to laboratories with limited equipment. However, with a highly automated system and the capacity to test up to 94 specimens within two hours (not including nucleic acid extraction and hands-on time), this high throughput Luminex RVP FAST method would be useful in large hospitals where they could have high input of respiratory samples to run by batch.

Globally, the fear of clinical worsening for patients with ARI usually results in empiric antibiotic prescription, even though doctors are aware that most ARI are caused by viruses. One of the factors contributing to this is the long interval between sampling and reporting of test results. The Luminex assay may be part of the solution to this with its fast turnaround time.

The Luminex has a number of weaknesses: it cannot distinguish enterovirus and rhinovirus, it is not quantitative, it comprises a two-tube step for RT-PCR and DNA hybridization, including an open-tube step for transferring PCR product from RT-PCR tube to hybridization tube, which brings a risk for contamination, and it is expensive. A significantly lower positivity rate of the Luminex assay for hBoV found in this study ( *P*=0.04) is also a weakness of this kit, which may need further clinical evaluation.

Limitations of this study are the lack of a true gold standard as is commonly seen when evaluating diagnostic assays and low numbers of positive samples for several ‘uncommon’ viruses (such as PIV1, PIV2). This low number of positive samples may conceal true diagnostic rates for these viruses.

In conclusion, our study shows the Luminex RVP FAST has a good diagnostic performance for detection of multiple respiratory viruses. Results from this study provided an additional evaluation on the utility of this commercial test. Though the cost of Luminex assay is rather high, Luminex RVP FAST platform could become affordable in large hospitals where samples are high input, reducing cost of the test by batch run. Once the per assay cost of this assay become more affordable, the above advantages and the short turnaround time could contribute to improving patient management and changing the prescription culture in countries like Vietnam.

## Data availability

***Figshare:*** Evaluation of the Luminex xTAG Respiratory Viral Panel FAST v2 assay for detection of multiple respiratory viral pathogens in nasal and throat swabs in Vietnam

**Dataset 1: Respiratory Viral Diagnostic Result from 442 patient swabs of the Luminex and reference assays.**

https://doi.org/10.6084/m9.figshare.5353630.v1 ^27^

## References

[1] Rudan I Boschi-Pinto C Biloglav Z: Epidemiology and etiology of childhood pneumonia. *Bull World Health Organ.* 2008;86(5):408–416. 10.2471/BLT.07.048769 18545744 PMC2647437

[2] Williams BG Gouws E Boschi-Pinto C: Estimates of world-wide distribution of child deaths from acute respiratory infections. *Lancet Infect Dis.* 2002;2(1):25–32. 10.1016/S1473-3099(01)00170-0 11892493

[3] Anders KL Nguyen HL Nguyen NM: Epidemiology and virology of acute respiratory infections during the first year of life: a birth cohort study in Vietnam. *Pediatr Infect Dis J.* 2015;34(4):361–370. 10.1097/INF.0000000000000643 25674708 PMC4418783

[4] van Gageldonk-Lafeber AB Heijnen ML Bartelds AI: A case-control study of acute respiratory tract infection in general practice patients in The Netherlands. *Clin Infect Dis.* 2005;41(4):490–497. 10.1086/431982 16028157 PMC7107976

[5] Chou CA Lin TI Chen YS: Comparisons of etiology and diagnostic tools of lower respiratory tract infections in hospitalized young children in Southern Taiwan in two seasons. *J Microbiol Immunol Infect.* 2016;49(4):539–545. 10.1016/j.jmii.2014.08.029 25442857

[6] Moesker FM van Kampen JJ van Rossum AM: Viruses as Sole Causative Agents of Severe Acute Respiratory Tract Infections in Children. *PLoS One.* 2016;11(3):e0150776. 10.1371/journal.pone.0150776 26964038 PMC4786225

[7] Fujitsuka A Tsukagoshi H Arakawa M: A molecular epidemiological study of respiratory viruses detected in Japanese children with acute wheezing illness. *BMC Infect Dis.* 2011;11:168. 10.1186/1471-2334-11-168 21663657 PMC3123215

[8] Mishra P Nayak L Das RR: Viral Agents Causing Acute Respiratory Infections in Children under Five: A Study from Eastern India. *Int J Pediatr.* 2016;2016; 7235482. 10.1155/2016/7235482 28018433 PMC5149672

[9] Liao RS Tomalty LL Majury A: Comparison of viral isolation and multiplex real-time reverse transcription-PCR for confirmation of respiratory syncytial virus and influenza virus detection by antigen immunoassays. *J Clin Microbiol.* 2009;47(3):527–532. 10.1128/JCM.01213-08 19129410 PMC2650906

[10] Advani S Sengupta A Forman M: Detecting respiratory viruses in asymptomatic children. *Pediatr Infect Dis J.* 2012;31(12):1221–1226. 10.1097/INF.0b013e318265a804 22739572 PMC3505556

[11] Aguilar JC Pérez-Breña MP Garcia ML: Detection and identification of human parainfluenza viruses 1, 2, 3, and 4 in clinical samples of pediatric patients by multiplex reverse transcription-PCR. *J Clin Microbiol.* 2000;38(3):1191–1195. 10699020 10.1128/jcm.38.3.1191-1195.2000 PMC86373

[12] Balada-Llasat JM LaRue H Kelly C: Evaluation of commercial ResPlex II v2.0, MultiCode-PLx, and xTAG respiratory viral panels for the diagnosis of respiratory viral infections in adults. *J Clin Virol.* 2011;50(1):42–45. 10.1016/j.jcv.2010.09.022 21050809 PMC7185502

[13] Mahony JB Blackhouse G Babwah J: Cost analysis of multiplex PCR testing for diagnosing respiratory virus infections. *J Clin Microbiol.* 2009;47(9):2812–2817. 10.1128/JCM.00556-09 19571025 PMC2738055

[14] Freymuth F Vabret A Cuvillon-Nimal D: Comparison of multiplex PCR assays and conventional techniques for the diagnostic of respiratory virus infections in children admitted to hospital with an acute respiratory illness. *J Med Virol.* 2006;78(11):1498–1504. 10.1002/jmv.20725 16998894 PMC7159369

[15] Gadsby NJ Hardie A Claas EC: Comparison of the Luminex Respiratory Virus Panel fast assay with in-house real-time PCR for respiratory viral infection diagnosis. *J Clin Microbiol.* 2010;48(6):2213–2216. 10.1128/JCM.02446-09 20357215 PMC2884497

[16] Popowitch EB O'Neill SS Miller MB: Comparison of the Biofire FilmArray RP, Genmark eSensor RVP, Luminex xTAG RVPv1, and Luminex xTAG RVP fast multiplex assays for detection of respiratory viruses. *J Clin Microbiol.* 2013;51(5):1528–1533. 10.1128/JCM.03368-12 23486707 PMC3647947

[17] Pabbaraju K Wong S Tokaryk KL: Comparison of the Luminex xTAG respiratory viral panel with xTAG respiratory viral panel fast for diagnosis of respiratory virus infections. *J Clin Microbiol.* 2011;49(5):1738–1744. 10.1128/JCM.02090-10 21411570 PMC3122679

[18] Salez N Vabret A Leruez-Ville M: Evaluation of Four Commercial Multiplex Molecular Tests for the Diagnosis of Acute Respiratory Infections. *PLoS One.* 2015;10(6):e0130378. 10.1371/journal.pone.0130378 26107509 PMC4481272

[19] Choudhary ML Anand SP Tikhe SA: Comparison of the conventional multiplex RT-PCR, real time RT-PCR and Luminex xTAG® RVP fast assay for the detection of respiratory viruses. *J Med Virol.* 2016;88(1):51–57. 10.1002/jmv.24299 26100490 PMC7166673

[20] Rabaa MA Tue NT Phuc TM: The Vietnam Initiative on Zoonotic Infections (VIZIONS): A Strategic Approach to Studying Emerging Zoonotic Infectious Diseases. *Ecohealth.* 2015;12(4):726–735. 10.1007/s10393-015-1061-0 26403795 PMC4700077

[21] Do LA van Doorn HR Bryant JE: A sensitive real-time PCR for detection and subgrouping of human respiratory syncytial virus. *J Virol Methods.* 2012;179(1):250–255. 10.1016/j.jviromet.2011.11.012 22119628 PMC3405522

[22] Jansen RR Schinkel J Koekkoek S: Development and evaluation of a four-tube real time multiplex PCR assay covering fourteen respiratory viruses, and comparison to its corresponding single target counterparts. *J Clin Virol.* 2011;51(3):179–185. 10.1016/j.jcv.2011.04.010 21571585 PMC7108253

[23] Chen JH Lam HY Yip CC: Clinical Evaluation of the New High-Throughput Luminex NxTAG Respiratory Pathogen Panel Assay for Multiplex Respiratory Pathogen Detection. *J Clin Microbiol.* 2016;54(7):1820–1825. 10.1128/JCM.00517-16 27122380 PMC4922091

[24] Jokela P Piiparinen H Mannonen L: Performance of the Luminex xTAG Respiratory Viral Panel Fast in a clinical laboratory setting. *J Virol Methods.* 2012;182(1–2):82–86. 10.1016/j.jviromet.2012.03.015 22465255 PMC7119588

[25] Tang YW Gonsalves S Sun JY: Clinical Evaluation of the Luminex NxTAG Respiratory Pathogen Panel. *J Clin Microbiol.* 2016;54(7):1912–1914. 10.1128/JCM.00482-16 27122378 PMC4922127

[26] Beckmann C Hirsch HH: Comparing Luminex NxTAG-Respiratory Pathogen Panel and RespiFinder-22 for multiplex detection of respiratory pathogens. *J Med Virol.* 2016;88(8):1319–1324. 10.1002/jmv.24492 26856438 PMC7166946

[27] Hang VTT Ny NTH Phuc TM: Dataset 1: Evaluation of the Luminex xTAG Respiratory Viral Panel FAST v2 assay for detection of multiple respiratory viral pathogens in nasal and throat swabs in Vietnam. *figshare.* 2017 Data Source 10.12688/wellcomeopenres.12429.2 PMC5811805 29503874

