# Supplementary material for: Evaluation of the Luminex xTAG Respiratory Viral Panel FAST v2 assay for detection of multiple respiratory viral pathogens in nasal and throat swabs in Vietnam
Source: Wellcome Open Res. 2018 Apr 30;2:80. Originally published 2017 Sep 5. [Version 2] doi: 10.12688/wellcomeopenres.12429.2 (PMC5811805; doi:10.12688/wellcomeopenres.12429.2)
Supplement: Peer review history

# Peer review: Referee response for version 1

**DOI:** 10.21956/wellcomeopenres.13459.r30347

**Published:** 2018-02-12

**Authors:** Marta Canuti

**Affiliations:** 1 Department of Biology, Memorial University of Newfoundland, St. John's, NL, Canada

Thi Ty Hang et al. evaluated the Luminex system’s performance in diagnosing respiratory viral infections in ARI cases in Vietnam and compared their results to those obtained with other reference diagnostic methods. Although more expensive than in-house diagnostic assays, Luminex performed well and it could be of real benefit in laboratories with a high throughput of samples.  

 The paper is well written, sample size is appropriate and conclusions are supported by the results. I only have minor suggestions.

1. I think Table 2 is a bit difficult to follow. When I read “positive rate” I think about N+/total number of test (while here it is over the N of positives or the N of negatives with the reference method – columns 6 and 7). I think rephrasing the captions for columns 6 and 7 and/or including a note with the used formulas will greatly increase the readability of the table (e.g. something like “Luminex sensitivity”, L+and Ref+/ Ref+ for column 6 and “additional Luminex positive”, L+/Ref- for column 7). Personally, I would also remove column 5, because it adds confusion.
2. Abstract, results: Similarly, the sentence “viral pathogens were detected in a total count of 270/294 by the Luminex among reference assays” is not clear.
3. Results, third paragraph: I would rephrase the sentence “…corresponding to a 1.8% detection rate” into something like “….corresponding to an additional 1.8% positives”. (Same at the following line).  
4. Methods: Was the used VTM commercially available or prepared in house? A reference to the provider or a list of component used for its preparation should be included.
5. Results, first paragraph: Shouldn’t it be <=15, instead of >=15?
6. Was the higher number of co-infections detected by the Luminex due to the higher number of Entero/Rhinoviruses identified?
7. Results, paragraph starting with “For individual targets”: I think a word is missing after “more often”.
8. Discussion: I would avoid the repetition of the K values as they are stated 2 paragraphs above.

I have read this submission. I believe that I have an appropriate level of expertise to confirm that it is of an acceptable scientific standard.

# Peer review: Referee response for version 1

**DOI:** 10.21956/wellcomeopenres.13459.r29351

**Published:** 2017-12-28

**Authors:** Bharti Malhotra

**Affiliations:** 1 Sawai Man Singh Medical College, Jaipur, Rajasthan, India

Approved with minor modifications, authors to address the following

1. Authors should provide sensitivity & specificity, limit of detection of the reference assay used
2. Authors should provide how the sample size was achieved
3. Was any other reference method used to assess whether additional positive by Luminex were false positive or true positive to truly evaluate the performance of luminex / label it as more sensitive

I have read this submission. I believe that I have an appropriate level of expertise to confirm that it is of an acceptable scientific standard.
